# Cost-Effectiveness Analysis of Digital Breast Tomosynthesis and Mammography in Breast Cancer Screening: A Markov Modeling Study

**DOI:** 10.1007/s44197-024-00239-z

**Published:** 2024-05-15

**Authors:** Wei-Shiuan Chung, Thomas T. H. Wan, Yu Tsz Shiu, Hon-Yi Shi

**Affiliations:** 1 Department of Medical Imaging, Kaohsiung Municipal Siaogang Hospital, Kaohsiung Medical University, Kaohsiung, Taiwan; 2 Department of Medical Imaging, Kaohsiung Medical University Hospital, Kaohsiung Medical University, Kaohsiung, Taiwan; 3 School of Global Health Management and Informatics, University of Central Florida, Orlando, USA; 4 Department of Healthcare Administration and Medical Informatics, Kaohsiung Medical University, No. 100, Tzyou 1st Road, Kaohsiung, Taiwan; 5 Department of Business Management, National Sun Yat-Sen University, Kaohsiung, Taiwan; 6 Department of Medical Research, Kaohsiung Medical University Hospital, Kaohsiung, Taiwan; 7 Department of Medical Research, China Medical University Hospital, China Medical University, Taichung, Taiwan

**Keywords:** Breast cancer screening, Mammography, Digital breast tomosynthesis, Cost-utility analysis, Markov model

## Abstract

**Background:**

Mammography (MG) has demonstrated its effectiveness in diminishing mortality and advanced-stage breast cancer incidences in breast screening initiatives. Notably, research has accentuated the superior diagnostic efficacy and cost-effectiveness of digital breast tomosynthesis (DBT). However, the scope of evidence validating the cost-effectiveness of DBT remains limited, prompting a requisite for more comprehensive investigation. The present study aimed to rigorously evaluate the cost-effectiveness of DBT plus MG (DBT-MG) compared to MG alone within the framework of Taiwan’s National Health Insurance program.

**Methods:**

All parameters for the Markov decision tree model, encompassing event probabilities, costs, and utilities (quality-adjusted life years, QALYs), were sourced from reputable literature, expert opinions, and official records. With 10,000 iterations, a 2-year cycle length, a 30-year time horizon, and a 2% annual discount rate, the analysis determined the incremental cost-effectiveness ratio (ICER) to compare the cost-effectiveness of the two screening methods. Probabilistic and one-way sensitivity analyses were also conducted to demonstrate the robustness of findings.

**Results:**

The ICER of DBT-MG compared to MG was US$5971.5764/QALYs. At a willingness-to-pay (WTP) threshold of US$33,004 (Gross Domestic Product of Taiwan in 2021) per QALY, more than 98% of the probabilistic simulations favored adopting DBT-MG versus MG. The one-way sensitivity analysis also shows that the ICER depended heavily on recall rates, biopsy rates, and positive predictive value (PPV2).

**Conclusion:**

DBT-MG shows enhanced diagnostic efficacy, potentially diminishing recall costs. While exhibiting a higher biopsy rate, DBT-MG aids in the detection of early-stage breast cancers, reduces recall rates, and exhibits notably superior cost-effectiveness.

## Introduction

Recent studies support mammography’s (MG) effectiveness in reducing breast cancer mortality and late-stage cases during screening [1–3]. High breast density, found in 50% of Western and 70% of Asian women, can limit MG’s sensitivity and cancer detection [4, 5]. To address this, additional screening tools like digital breast tomosynthesis (DBT) have emerged, offering potential benefits such as reduced false positives, improved cancer detection, and fewer recalls [6–8]. This highlights the evolving nature of breast cancer screening and management.

The DBT is gaining recognition for its improved cancer detection abilities, particularly in women with dense breast tissue [9]. Evidence from the ASTOUND-2 trial, which focused on women with dense breasts and digital MG, revealed an incremental cancer detection rate of 2.83 per 1000 screens [10]. Additionally, a meta-analysis study reported an incremental cancer detection rate of 2.4 cancers per 1000 screens in biennial screening using DBT, with only a marginal increase in recall rates compared to MG [2]. However, limited research has examined the cost-effectiveness of DBT plus MG compared to MG in the context of healthcare economics. Although some studies have compared their cost-effectiveness, only a handful have delved into aspects like recall rates and the broader economic impact, including productivity [9, 11–14]. Importantly, cost-effectiveness and utility analyses of these screening methods within Asian populations and Taiwan’s National Health Insurance (NHI) program are notably lacking. Therefore, this study aims to fill this gap by investigating the cost-effectiveness of DBT plus MG (DBT-MG) compared to MG alone in breast cancer screening in Taiwan.

## Materials and Methods

### Research Design

From the perspective of the Taiwanese Government, which encompasses a comprehensive societal viewpoint accounting for both direct and indirect costs, including productivity losses. The Markov model was employed to perform dynamic simulations and estimations based on existing literatures [15–19]. Then, we analyzed the recall rate, cancer detection rate, breast cancer detection rate, treatment cost of each stage, breast cancer and related quality-adjusted life years (QALYs), and other related data. Specifically, the Markov decision model were used to simulate 10,000 times for every two-year cycle. This study was reviewed and approved by the Institutional Review Board of the Kaohsiung Medical University Hospital (KMUHIRB-E(I)-20220012).

### Markov Decision Modeling Assumptions

**Fig. 1:**
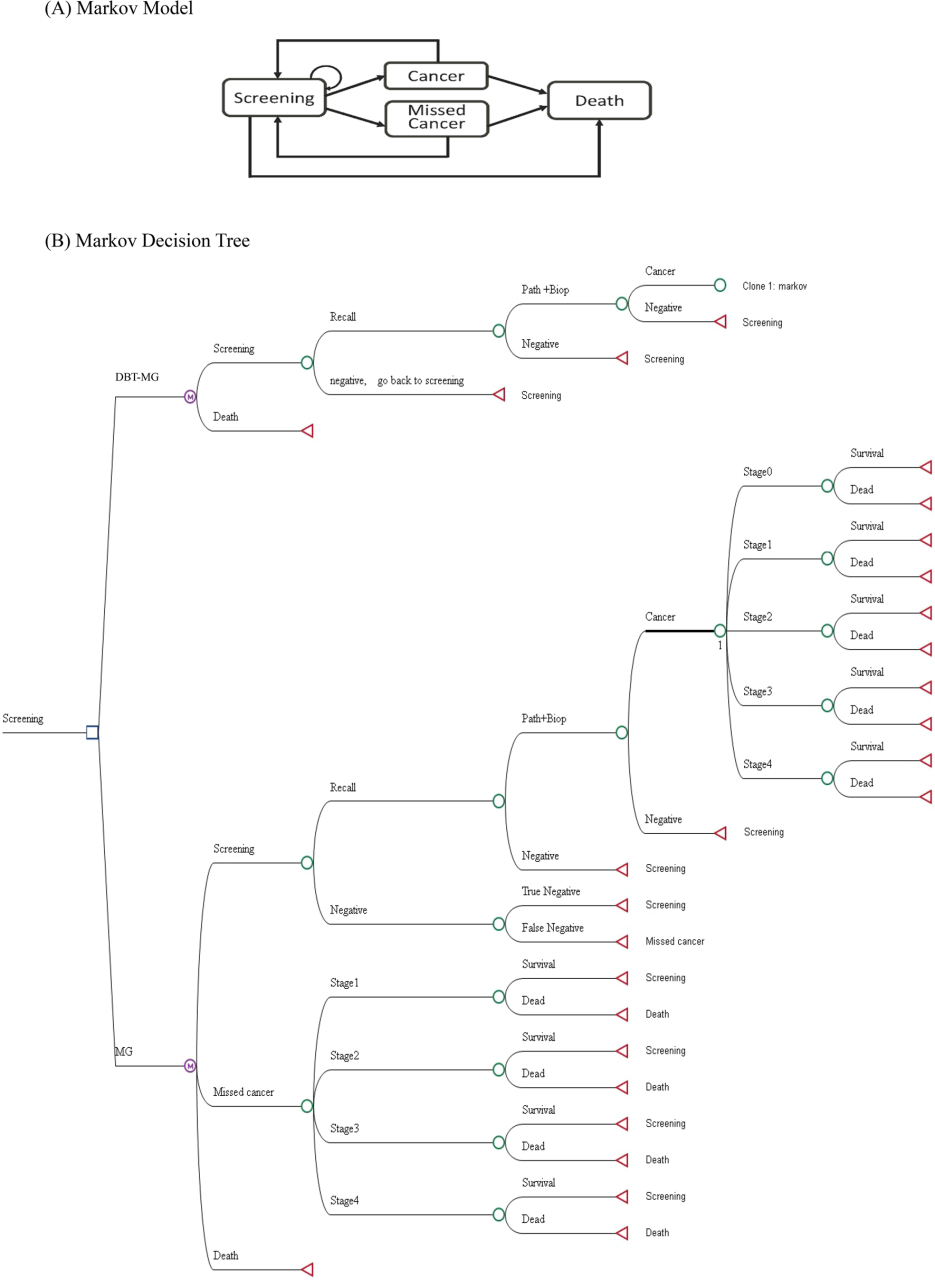
Cost-effectiveness analysis of digital breast tomosynthesis (DBT) plus mammography (MG) compared to MG in breast cancer screening. **A** Markov model. **B** Markov decision tree

Informed by clinical practices in Taiwan and relevant literature, we developed the status transition diagram of the Markov model utilized in this study (Fig. 1A). The model delineates four states after entering the screening cycle: screening, cancer, missed cancer, and death. In the international medical context, Taiwan’s postoperative or post-treatment breast cancer surveillance strategies advocate ongoing screening [15, 20, 21]. Following breast cancer surgery, patients who have undergone partial or unilateral mastectomy are advised to continue breast screening, although the recommended screening intervals may vary by country and individual case. In addition to biennial breast screening, diagnostic mammography may also be necessary. Our study assumes that all patients will adhere to the recommendation for continued breast imaging examinations following treatment. Additionally, the selection of a 30-year time horizon for our economic evaluation was predicated on Taiwan’s existing breast screening policy, which mandates biennial screenings. This temporal scope captures the period of screening eligibility and accommodates mortality from breast cancer and other causes. In adherence to this policy, all asymptomatic individuals, irrespective of cancer detection status, undergo biennial screening until the conclusion of their lifespans. Natural mortality rates for both screening tools (DBT-MG & MG) were presumed equal for each cycle and were not factored into the model; only breast cancer-related mortality was simulated [15]. Upon transitioning to the death stage, individuals remain in this state indefinitely, precluding further transitions.

The Markov decision tree model is illustrated in Fig. 1B. Subsequent to screening, outcomes encompass negative results or recalls. Upon recall, results may be negative or necessitate further pathologic diagnosis (path + biop), potentially culminating in a negative breast cancer pathology result. Our premise posits that all asymptomatic screening subjects, irrespective of cancer detection, perpetuate the biennial screening cycle until mortality. Moreover, we posit an absence of post-screening loss to follow-up or untreated individuals in Breast Imaging Reporting and Data System (BI-RADS) categories 0, 4, and 5. Such subjects would undergo subsequent evaluation [22, 23]. To streamline model intricacies, false-negative outcomes with MG are construed as missed cancers. Furthermore, “missed cancer” refers to instances where cancer remains undetected despite the utilization of both DBT-MG and MG screening modalities. It is quantified by the difference in cancer detection rates between the DBT-MG and MG groups [24]. Therefore, in the present study, the DBT-MG group is not included in the category of “missed cancer.” Additionally, in consonance with disparities in cancer detection rates between DBT-MG and MG, we presuppose the absence of stage 0 breast cancer occurrences among missed cancers using MG; other stages (1–4) are redistributed based on national incidence rates. Enhanced screening tools tend to discern a greater proportion of early-stage cancers, potentially yielding cost efficiencies and enhanced clinical outcomes. Breast cancer stages 0 through 4 are encompassed within the model. Following each two-year cycle culmination, in instances where screening results, reexamination, biopsy, and subsequent stages yield negative findings, or where no breast cancer-related mortalities occur, the cycle reverts to breast cancer screening status.

### Transitional Model Parameters

#### Probability

This study adopted Pan et al. [15] as the primary basis of the screening effectiveness of DBT-MG and MG, such as recall rate, cancer detection rate, biopsy rate, and biopsy positive rate. To our knowledge, this is the first study to compare the results before and after the complete introduction of DBT-MG to replace MG as the breast screening tool in a medical center in Taiwan. DBT-MG and MG were used to diagnose breast cancer at each stage, and the study by Pan et al. [15] calculated the ratio of missed cancer by MG at each stage and the publicly available data of the Health Promotion Administration (Cancer Registry Annual Report 2019), respectively [16]. We assumed that there were no stage 0 breast cancer cases among the missed cancers using MG, and the remaining stages (stages 1–4) were redistributed based on the national incidence (Table 1).

**Table 1:** Key inputs in the model

Transition probabilities	Parameters	Distribution	Literatures/sources
DBT-MG recall rate	9.00%	β	Pan et al. [15]
MG recall rate	11.81%	β	Pan et al. [15]
DBT-MG cancer detection rate	8.68‰	β	Pan et al. [15]
MG cancer detection rate	7.55‰	β	Pan et al. [15]
DBT-MG slice rate	34.69%	β	Pan et al. [15]
MG slice rate	20.56%	β	Pan et al. [15]
DBT-MG slice positive rate (PPV2)	27.80%	β	Pan et al. [15]
MG slice positive rate (PPV2)	31.10%	β	Pan et al. [15]
Probability of survival rate of stage 0	97.70%	β	MOHW [16]
Probability of survival rate of stage I	95.70%	β	MOHW [16]
Probability of survival rate of stage II	89.10%	β	MOHW [16]
Probability of survival rate of stage III	72.30%	β	MOHW [16]
Probability of survival rate of stage IV	25.70%	β	MOHW [16]
DBT-MG diagnosis rate of stage 0	30.40%	β	Pan et al. [15]
DBT-MG diagnosis rate of stage I	31.54%	β	Pan et al. [15] & MOHW [16]
DBT-MG diagnosis rate of stage II	23.94%	β	Pan et al. [15] & MOHW [16]
DBT-MG diagnosis rate of stage III	8.73%	β	Pan et al. [15] & MOHW [16]
DBT-MG diagnosis rate of stage IV	5.39%	β	Pan et al. [15] & MOHW [16]
MG diagnosis rate of stage 0	18.30%	β	Pan et al. [15] & MOHW [16]
MG diagnosis rate of stage I	31.09%	β	Pan et al. [15] & MOHW [16]
MG diagnosis rate of stage II	31.83%	β	Pan et al. [15] & MOHW [16]
MG diagnosis rate of stage III	11.61%	β	Pan et al. [15] & MOHW [16]
MG diagnosis rate of stage IV	7.17%	β	Pan et al. [15] & MOHW [16]
MG no diagnosis rate of stage 0	0.00%	β	Assumption
MG no diagnosis rate of stage I	38.05%	β	MOHW [16]
MG no diagnosis rate of stage II	38.96%	β	MOHW [16]
MG no diagnosis rate of stage III	14.21%	β	MOHW [16]
MG no diagnosis rate of stage IV	8.77%	β	MOHW [16]

Costs ($)	Parameters	Distribution	Literatures/sources
Direct cost			
DBT-MG examination	150.00	γ	MOHW [16]
MG examination	41.50	γ	MOHW [16]
Re-clinical visits	65.38	γ	MOHW [16]
Biopsy and pathological diagnosis	525.67	γ	MOHW [16]
Treatment for stage 0	6202.80	γ	Wang et al. [17]
Treatment for stage I	76,506.48	γ	Wang et al. [17]
Treatment for stage II	82,421.77	γ	Wang et al. [17]
Treatment for stage III	93,272.99	γ	Wang et al. [17]
Treatment for stage IV	69,446.35	γ	Wang et al. [17]
Death	0.00		Assumption
Indirect cost			
Re-clinical visits	43.83	γ	MOHW [16]
Biopsy and pathological diagnosis	41.74	γ	MOHW [16]
Treatment for stage 0	609.64	γ	Huang et al. [18]
Treatment for stage I	609.64	γ	Huang et al. [18]
Treatment for stage II	609.64	γ	Huang et al. [18]
Treatment for stage III	609.64	γ	Huang et al. [18]
Treatment for stage IV	609.64	γ	Huang et al. [18]
Death	5305.34	γ	Huang et al. [18]

Utilities (QALYs)	Parameters	Distribution	Literatures/sources
Within first year QALYs for stage 0	0.904	γ	Schousboe et al. [19]
After first year QALYs for stage 0	1.000	γ	Schousboe et al. [19]
Within first year QALYs for stage I	0.846	γ	Schousboe et al. [19]
After first year QALYs for stage I	0.985	γ	Schousboe et al. [19]
Within first year QALYs for stage II	0.846	γ	Schousboe et al. [19]
After first year QALYs for stage II	0.985	γ	Schousboe et al. [19]
Within first year QALYs for stage III	0.753	γ	Schousboe et al. [19]
After first year QALYs for stage III	0.932	γ	Schousboe et al. [19]
Within first year QALYs for stage IV	0.753	γ	Schousboe et al. [19]
After first year QALYs for stage IV	0.832	γ	Schousboe et al. [19]
QALYs for no breast cancer	1.000		Assumption
QALYs for death	0.000		Assumption

*DBT* Digital breast tomosynthesis; *MG* mammography; *QALYs* quality-adjusted life years

#### Cost

Initially, both direct and indirect costs were computed using an exchange rate of 30:1 for NTD to USD. Subsequently, the direct costs and indirect costs were determined based on the actual treatments received by patients and the reimbursements from the national health insurance, adjusted to 2021 prices using the consumer price index (CPI). Direct costs included direct medical costs of screening, recall, biopsy, and pathological diagnosis, as well as direct medical costs associated with different stages of cancer, as described below. (1) Direct costs of examination: initial examination cost for DBT-MG was $150, whereas MG cost $41.5. The DBT-MG cost was derived from the current self-funded examination rate approved by the Kaohsiung Municipal Government ($150), while the MG cost represented the health insurance reimbursement rate for MG ($41.5). In general, the total cost per screening before being diagnosed with breast cancer was $177.81 for DBT-MG and $171.39 for MG, with a difference of only $6.43. (2) Direct costs of recall: health insurance costs of recall included $588 for breast ultrasonography, $286 for general outpatient diagnosis, and $1374 for diagnostic MG. Based on clinical experience and the health insurance payment review procedures and amendments, we assumed that each recalled screenee would undergo ultrasound reexamination twice within two years, and one in 20 recalled patients would undergo diagnostic MG reexamination four times over two years, etc. (3) Direct costs of biopsy and pathological diagnosis: $526 would be paid in accordance with the payment criteria for the diagnosis of breast cancer according to the Breast Cancer Medical Payment Improvement Program of National Health Insurance (revised on July 1st, 2021). (4) Direct costs associated with different stages of cancer: according to Lin et al. [25], in Taiwan, the lifetime medical expenditures (LME) of breast cancer in different stages were calculated based on the Health Promotion Administration and National Health Insurance Administration data. We hypothesize that due to the lower five-year survival rate and shorter life expectancy associated with Stage 4, the potential medical expenses incurred may also be lower compared to Stage 3.

Additionally, indirect costs included the indirect social costs of recall, biopsy, and pathological diagnosis, as well as treatment at different stages of cancer, as described below. (1) Indirect costs of recall, biopsy, and pathological diagnosis: we assumed that half a day (four hours) of productivity would be lost for a subsequent visit due to recall, and one day (eight hours) of productivity would be lost for pathological diagnosis and biopsy. According to the Employee Salary Survey and Labor Force Participation Rate in Taiwan announced by the Directorate General of Budget, Accounting, and Statistics, the Executive Yuan in 2021 [26], the hourly wage for women was $10, and the average labor force participation rate was 51.49%. The indirect costs of recall and biopsy diagnosis were calculated using the equation “lost labor force hours × hourly wage × labor force participation rate.” (2) Indirect costs of breast cancer at different stages and death: based on Huang et al. study conducted in Taiwan, the three-year indirect costs of breast cancer morbidity and mortality were predicted to be $588 and $5117, respectively [18].

#### Effectiveness (QALYs)

A QALY quantifies a year lived in perfect health as 1, calculated by multiplying the duration in a health state by its utility value. This study assumed that the QALYs of the screenee with breast cancer was 1 and that of the deceased was 0. Since there were no suitable studies in Taiwan regarding the QALYs of breast cancer for reference, we adopted the QALYs of the first year and beyond at each stage of breast cancer based on an American study conducted by Schousboe et al. [19]. Then, we calculated the utility loss of each cycle (two years per cycle) relative to healthy patients and input the data into the Markov decision model for simulation.

### Cost-Utility Analysis (CUA)

CUA was used to compare the difference in total medical costs and QALYs between the two screening options, and the results were expressed as an incremental cost-effectiveness ratio (ICER). First, we established the Markov decision model, and each state transition probability value, cost, and effectiveness/utility value were pre-set. Second, the results of each stage were summed by simulating the state transition of patients during the course of treatment. The Markov decision tree model was used to simulate the understanding of which option is more cost-effective. Additionally, in the Markov decision model, the ICER of the two screening tools was simulated using a 2% annual discount rate in cost and QALYs based on the literature and willingness to pay (WTP) was set as gross domestic product (GDP) per capita in 2022, namely, $33,004.

### Sensitivity Analysis

The sensitivity analysis was conducted to identify which risk or uncertainty sources have the most significant potential impact on breast cancer. The descriptions of related settings are as follows: (1) one-way sensitivity analysis: with all uncertainties maintained under baseline conditions, the degree to which the uncertainty of each factor affects the terminal objective analyzed. A tornado diagram was used as the analysis tool. All sensitive parameters were ranked according to their sensitivities to reflect the impact of each sensitive parameter on the result. The examination effects of the two screening tools in this study (recall rate, biopsy rate, positive predictive value, cancer detection rate, etc.) were measured with parameters presented in the range of the available national literature. Parameters, such as total medical costs and QALYs value of each stage and state underwent ± 20% variation based on the national data and literature. The results are presented in the tornado diagram. (2) Probabilistic sensitivity analysis (PSA): the model variables show a probability distribution. The parameters were randomly sampled using Monte Carlo simulation. The distribution of cost and utility in each quadrant was analyzed by the scatter plot. Additionally, the cost-utility benefits of DBT-MG became more pronounced with increasing screening cycles. However, the enhancement in cost-utility from 20 to 30 consecutive years was not as notable as from 10 to 20 consecutive years, suggesting a dose effect on the screening policy. It further underscores the importance of continued implementation of breast cancer screening policies to achieve better outcomes spontaneously. We also conduct the sensitivity analysis of different screening cycles of DBT-MG and MG for breast cancer screening, spanning 10-year, 20-year, and 30-year time horizons. WTP thresholds and ICERs were checked to determine whether the best breast screening option could be available under the highest cost-utility rate and within the acceptable payment range of screening subjects.

Descriptive and inferential statistical analyses were performed using IBM SPSS 23. The cost-effectiveness acceptability (CEA) and sensitivity analysis were performed using TreeAge Pro Healthcare 2021. Statistical significance was set at α = 0.05.

## Results

According to the results of the Markov decision model (Table 2), after 10,000 simulations and 15 cycles for a total of 30 years, the cumulative cost of DBT-MG was $7861.14, and MG was $7621.73. The incremental cost was $239.42. Regarding the effectiveness, DBT-MG was 22.9942 QALYs, and MG was 22.9541 QALYs. The incremental effectiveness was 0.0401 QALYs. The ICER of DBT-MG vs. MG was $5971.5764/QALYs, which was far lower than the set WTP. This finding suggests that applying DBT-MG is more cost-effective than MG to biennial breast screening. Additionally, the Markov cost-utility analysis results of five cycles (10 years), 10 cycles (20 years), and 15 cycles (30 years) were simulated with different screening years (Table 2). All ICERs were lower than the WTP for the screening cycles from 10 to 30 years. As the screening cycle increased, the cost-utility results of DBT-MG became more accentuated. However, the increase in cost utility from 20 to 30 consecutive years was not as significant as from 10 to 20 consecutive years.

**Table 2:** Sensitivity analysis compared the screening cycles of digital breast tomosynthesis (DBT) combined with mammography (MG) (DBT-MG) versus MG alone in breast cancer screening

Time horizon	Total cycle*	Strategy	Total costs ($)	Incremental costs ($)	Effectiveness (QALYs)	Incremental effectiveness (QALYs)	ICUR ($/QALYs)	Probability sensitivity analysis (PSA) (%)
10 years	5 times	DBT-MG	3159.16	150.76	9.2405	0.0103	14,645.1705	81.41
		MG	3008.40		9.2302			
20 years	10 times	DBT-MG	5745.84	201.21	16.8037	0.0247	8132.0405	95.62
		MG	5544.63		16.7789			
30 years	15 times	DBT-MG	7861.14	239.42	22.9942	0.0401	5971.5764	98.48
		MG	7621.78		22.9541			

*QALYs* Quality-adjusted life years; *ICUR* incremental cost utility ratio

*Per cycle is two years

**Fig. 2:**
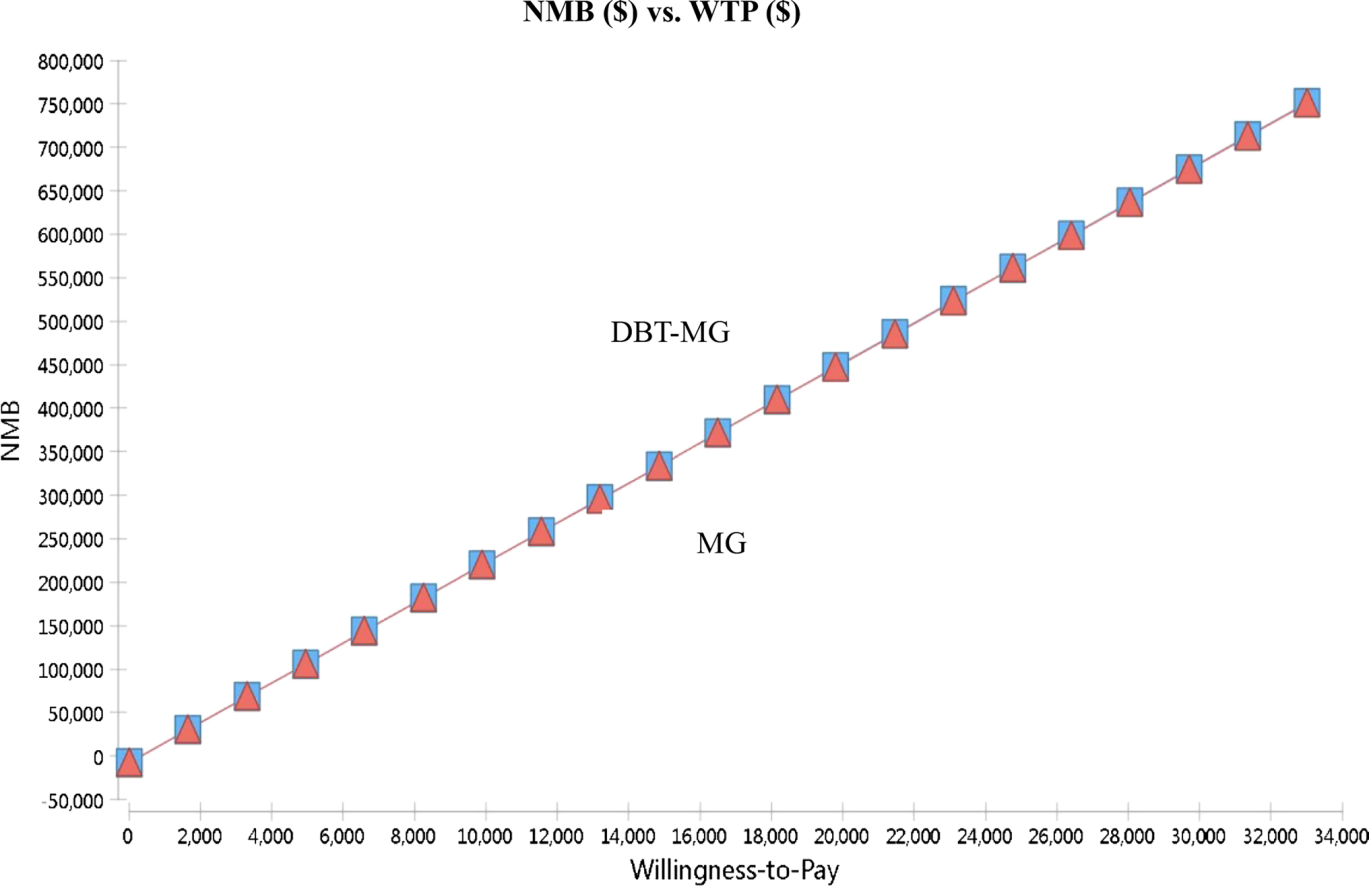
Net money benefit (NMB) of digital breast tomosynthesis (DBT) plus mammography (MG) compared to MG in breast cancer screening. *WTP* willingness-to-pay

In the comparative assessment of Net Monetary Benefits (NMBs) between the two screening tools, an incremental rise in the WTP threshold corresponded with an augmentation in the NMBs for both strategies (Fig. 2). Despite this trend, the differential magnitude between the two approaches remained relatively stable. Critically, the NMB associated with the DBT-MG exceeded that of the MG, underscoring the enhanced cost-utility of the integrated screening methodology.

**Fig. 3:**
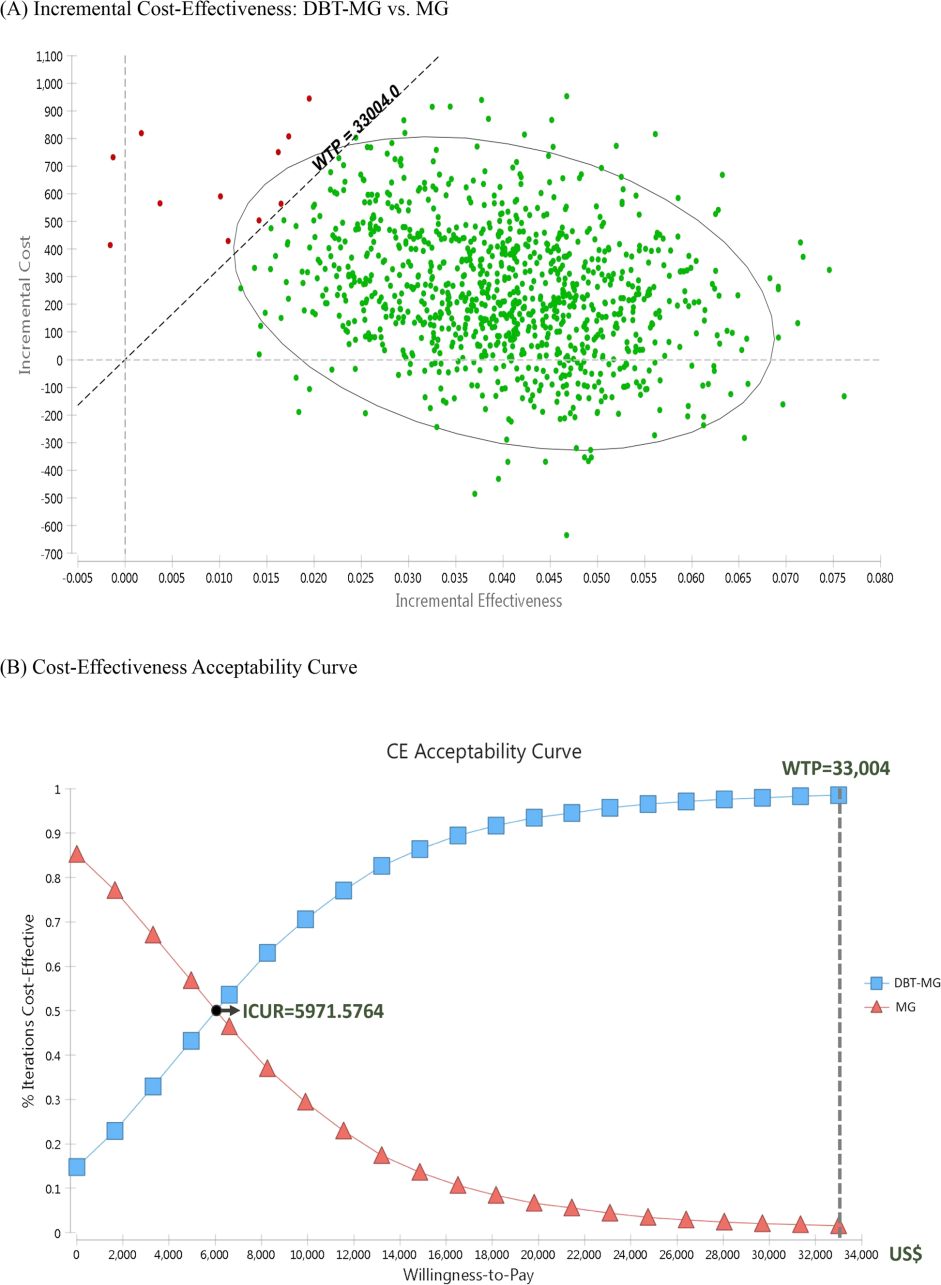
Probabilistic sensitivity analyses. **A** Incremental cost-effectiveness: DBT-MG vs. MG. Each green dot means an ICER. **B** Cost-effectiveness acceptability curve. *ICER* incremental cost effectiveness ratio; *DBT* digital breast tomosynthesis; *MG* mammography; *WTP* willingness-to-pay

After 10,000 Monte Carlo simulations, 10,000 points were presented in the incremental cost-utility scatter plot (Fig. 3A). The findings indicate an 83.7% probability that it is located in the cost-effective zone in the first quadrant (lower than the WTP); i.e., it is, therefore, cost-effective. Moreover, there was a 14.78% probability that it is located in the cost-saving zone of the fourth quadrant. Therefore, DBT-MG had a total probability of more than 98.48% as the more cost-effective breast screening option. Additionally, the results showed that the probability of the cost-effectiveness of MG decreases with an increasing WTP threshold, while that of DBT increase with an increasing WTP threshold (Fig. 3B). When the WTP is greater than the crossing WTP value of the acceptance curve of DBT-MG, which is $5971.5764/QALYs, the probability of the cost-effectiveness of DBT-MG is greater than that of MG.

**Fig. 4:**
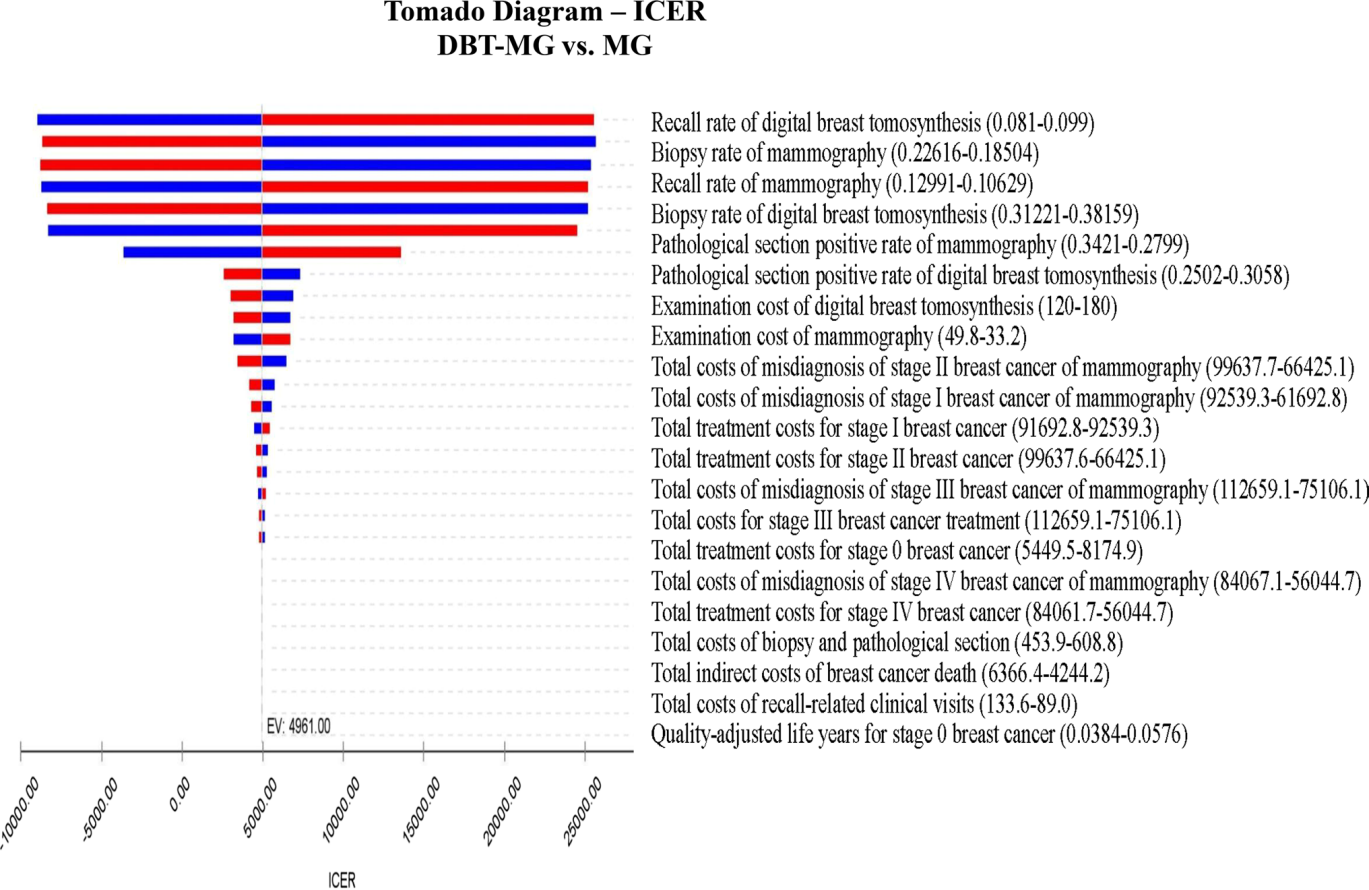
Deterministic sensitivity analysis—results as tornado plot. Bars indicate the effect of a ± 20% variance of a variable on the ICER. The red segments of the bars represent values that elevate the base case ICER, while the blue segments depict values that reduce the base case ICER. This color-coded scheme aids in interpreting the sensitivity analysis outcomes*. DBT* digital breast tomosynthesis; *MG* mammography; *ICER* incremental cost effectiveness ratio

In the context of cost and QALY estimations, input parameters were anchored to the central estimates of the Markov decision tree model, with a permissible range of ± 20%. An exhaustive one-way sensitivity analysis was conducted to evaluate the impact of individual variables on the ICER, as delineated in Fig. 4. The outcomes of this analysis demonstrated a spectrum of ICERs, spanning from − $9020.36/QALYs to $25660.23/QALYs. Notably, the most influential determinants on the ICERs encompassed the recall rate associated with DBT-MG, the pathological biopsy rate for MG, MG’s recall rate, the pathological biopsy rate for DBT-MG, the biopsy’s positive predictive value 2 (PPV2) for MG, and DBT-MG’s biopsy PPV2.

## Discussion

In Taiwan, despite the higher initial examination cost of DBT-MG compared to MG, DBT-MG demonstrates superior diagnostic capability, potentially reducing recall costs. Despite a higher biopsy rate, DBT-MG facilitates the detection of more breast cancers, particularly in the early stages. According to existing literature, early breast cancer treatment is associated with lower costs and higher QALYs. Thus, employing DBT-MG aids in the early detection and treatment of breast cancer, potentially reducing subsequent cumulative treatment costs and increasing cumulative QALYs. Simulation-based cost-utility analysis corroborates that while the cumulative cost of DBT-MG rises, cumulative QALYs also increase. With a probability exceeding 98.48% under the WTP, the 30-year simulation indicates that DBT-MG is likely to be cost-effective and cost-saving.

This Markov decision model includes screening, recall, and further pathologic diagnosis stages. The cost of treating breast cancer is high, but the actual cancer detection rate during screening is low (< 1%). Thus, cancer treatment costs have minimal impact in the overall model. The recall rate, biopsy rate, and biopsy positive rate significantly affect the model's outcomes, based on one-way sensitivity analysis.

The NMB metric amalgamates both costs and effectiveness into a unified measure, computed as the product of effectiveness and the WTP threshold, subtracted by costs. In the present study, as the WTP increased, the NMB of both screening strategies also increased. However, reviewing the data in detail, we found that when WTP was set as $33,004, DBT-MG had an NMB of $751,039.34, and MG had an NMB of $749,955.53. Therefore, DBT-MG has relative advantages over MG.

Our study is in line with prior research in the US and Europe, demonstrating the cost-effectiveness benefits of DBT-MG (Table 3) [9, 17, 24, 27, 28]. Notably, it’s the first to integrate societal-level indirect costs, including productivity loss, into the analysis. While data limitations exist, we used Taiwanese medical data supplemented by international literature, expert input, and some assumptions. Our Markov decision model's key parameters, such as transitional probabilities, costs, and QALYs, mainly relied on government sources and Taiwanese literature. These findings strongly support the government’s adoption of DBT-MG as a preferred breast screening policy [9, 17, 24, 27, 28].

**Table 3:** Highlights of the selected studies of cost effectiveness analysis of digital breast tomosynthesis (DBT) plus mmammography (MG) (DBT-MG) compared to MG alone in breast cancer screening

Authors (years)	Country/study design	Study subjects	Perspectives	WTP	Cost effectiveness	ICER/ICUR	Major findings
Present study	Taiwan/retrospective	40–69 years/screening every 2 years	Societal	$33,004/QALYs	DBT-MG	$5971.57/QALYs	ICUR is more correlated with recall rate and PPV2 as well as biopsy rate, and has a lower correlation with treatment and examination costs There is a probability of over 98% that DBT-MG compared to MG is more cost-effective or saving
Sankatsing et al. [9]	Netherlands/retrospective	50–74 years/screening every 2 years	–	€20,000 or €35,000/LYG	DBT-DM	€27,023/LYG	DBT screening led to incremental discounted lifetime effects of 5.09 LYGs and an increase in lifetime costs of €137 555 per 1000 women compared with DM The probability of DBT being more cost-effective was 0.36 at €20 000 and 0.66 at €35 000 per LYG
Lee et al. [27]	American/retrospective	50–74 years/dense breasts/screening every 2 years	Federal government payer	$100,000/QALYs	DBT-MG	$53,893/QALYs	For every 1000 screened individuals undergoing 12 rounds of DBT + MG screening, it can result in a reduction of 0.5 deaths and prevent 405 instances of false positive findings The excess cost of DBT has the greatest impact on cost-effectiveness
Kalra et al. [24]	American/ retrospective	40–79 years/screening every year	Federal government payer	$100,000/QALYs	DBT-MG	$20,300/QALYs	DBT is more cost-effective, and the ICER increases with age For the additional cost of DBT, when it is less than $250, DBT is more cost-effective
Cressman et al. [28]	Canada/retrospective	40–74 years/screening every 2 years	Government payer	C$ 100,000/QALYs	DBT-MG	C$17,149/QALYs	ICER is highly correlated with the reduction in recall rate, and it is less correlated with cancer detection rate There is a probability of over 95% that DBT is superior to MG
Wang et al. [17]	Netherlands/retrospective	50–75 years/screening every 2 years	–	–	DBT-MG	–	DBT is more cost-effective in women with dense breasts, but in the general population, the price of DBT plays a significant role in determining its cost-effectiveness

*PPV2* Positive predictive value for biopsies; *ICER* incremental cost effectiveness ratio; *ICUR* incremental cost utility ratio; *DBT* digital breast tomosynthesis; *LYGs* life years gained; *DM* digital mammography

In our sensitivity analysis, recall rate, PPV2, and biopsy rate were key factors influencing the ICER, with DBT-MG examination cost being less critical [9, 17, 24, 27, 28]. Probabilistic sensitivity analysis over 15 cycles and 30 years indicated a high likelihood (> 98.48%) of DBT-MG being cost-effective and cost-saving. Previous studies on breast screening tools’ cost-effectiveness vary, but collectively support DBT’s effectiveness. Our study shares similarities with previous work but offers unique insights.

Through further analysis using Pan et al. we found that the breast cancer detection rate of DBT-MG was 8.68‰, while that of MG was 7.55‰ [15]. The early breast cancer detection rate of DBT-MG (cancer detection rate * early breast cancer detection rate) was calculated as 5.31‰, while the early breast cancer detection rate of MG was 3.62‰. Therefore, DBT-MG can detect 1.69 more cases of early breast cancer than MG per 1000 women screened. The total cost per screening before being diagnosed with breast cancer was $177.81 for DBT-MG and $171.39 for MG, with a difference of only $6.43. Overall, if all the 738,974 women screened in 2019 had been screened with DBT-MG and had the same diagnostic capability advantages, the total costs would increase by $4,751,602.82 and about 1248 more women with early-stage breast cancer would be detected [16]. This analysis can also provide a reference for the government to decide on future screening policies.

This study has limitations. It relied on data from a single medical center in Taiwan for DBT-MG and MG [15], which may limit generalizability. Furthermore, some cost calculations were based on assumptions rather than real-world data. QALYs data specific to Taiwanese women is lacking. Additionally, important factors like age, breast density, health status, and family history were not extensively considered, which could be explored in future research.

## Conclusions

An effective breast screening tool is vital for early cancer detection and reducing the burdens of breast cancer [29]. This study suggests the government should prioritize using DBT-MG for screening, initially for high-risk groups, and then potentially for the entire population, based on the experience with MG screening since 2002. Our 30-year simulation with 15 cycles found DBT-MG to be highly cost-effective at $5971.57/QALYs, well below the WTP threshold. Wider DBT-MG adoption can enhance early breast cancer detection and program efficiency. Notably, the recall rate, biopsy rate, and biopsy positive rate PPV2 impact sensitivity. This study provides valuable insights for healthcare decision-makers and suggests analyzing cost-effectiveness for various risk groups and additional variables in future research.

## Data Availability

All data generated can be found in the published article. The data analyzed can be provided upon request.

## Abbreviations

MG: Mammography
DBT: Digital breast tomosynthesis
NHI: National health insurance
QALYs: Quality-adjusted life years
BI-RADS: Breast imaging reporting and data system
LME: Lifetime medical expenditures
ICER: Incremental cost effectiveness ratio
WTP: Willingness to pay
GDP: Gross domestic product
PSA: Probabilistic sensitivity analysis
CEA: Cost-effectiveness acceptability
NMB: Net monetary benefits
PPV2: Positive predictive value 2
